# Markedly Enhanced Surface Hydroxyl Groups of TiO_2_ Nanoparticles with Superior Water-Dispersibility for Photocatalysis

**DOI:** 10.3390/ma10050566

**Published:** 2017-05-22

**Authors:** Chung-Yi Wu, Kuan-Ju Tu, Jin-Pei Deng, Yu-Shiu Lo, Chien-Hou Wu

**Affiliations:** 1 Department of Biomedical Engineering and Environmental Sciences, College of Nuclear Science, National Tsing Hua University, Hsinchu 30013, Taiwan; s9812805@m98.nthu.edu.tw (C.-Y.W.); sacredbless@hotmail.com (K.-J.T.); yslo2001@gmail.com (Y.-S.L.); 2 Department of Chemistry, Tamkang University, Taipei 25137, Taiwan; jpdeng@mail.tku.edu.tw

**Keywords:** TiO_2_, alkaline hydrogen peroxide, terminal hydroxyl group, dispersion, dye sensitization

## Abstract

The benefits of increasing the number of surface hydroxyls on TiO_2_ nanoparticles (NPs) are known for environmental and energy applications; however, the roles of the hydroxyl groups have not been characterized and distinguished. Herein, TiO_2_ NPs with abundant surface hydroxyl groups were prepared using commercial titanium dioxide (ST-01) powder pretreated with alkaline hydrogen peroxide. Through this simple treatment, the pure anatase phase was retained with an average crystallite size of 5 nm and the surface hydroxyl group density was enhanced to 12.0 OH/nm^2^, estimated by thermogravimetric analysis, Fourier transform infrared spectroscopy, and X-ray photoelectron spectroscopy. Especially, this treatment increased the amounts of terminal hydroxyls five- to six-fold, which could raise the isoelectric point and the positive charges on the TiO_2_ surface in water. The photocatalytic efficiency of the obtained TiO_2_ NPs was investigated by the photodegradation of sulforhodamine B under visible light irradiation as a function of TiO_2_ content, pH of solution, and initial dye concentration. The high surface hydroxyl group density of TiO_2_ NPs can not only enhance water-dispersibility but also promote dye sensitization by generating more hydroxyl radicals.

## 1. Introduction

Nano-sized titania has emerged as one of the most fascinating materials in the fields of environmental remediation and energy conversion [1,2]. The surface hydroxyl group density of TiO_2_ is considered to play a significant role in the photocatalytic processes [3,4,5]. Surface hydroxylated centers have been proposed to act as trapping sites for both electrons and holes migrated to the surface [6,7,8]. Several investigations have demonstrated that the additional hydroxyl groups on the TiO_2_ surface can improve the adsorption capacity, the formation of mesoporous structure, and the catalytic efficiency [9,10,11]. However, most preparation methods for TiO_2_ with increased surface hydroxyl groups have complicated surface modification and are based on a sol-gel process from a precursor in special reaction conditions [12,13,14,15,16,17,18]. Each modification method may cause a more complex system, which is difficult to be generalized and each sol-gel process usually causes inconsistent results, which can hardly be used for industrial production.

The surface of TiO_2_ can react immediately with water molecules in either aqueous solutions or humid air, and hydroxyl groups are formed. When the surface of TiO_2_ is fully hydroxylated, the oxide ions in the oxide and water absorbed on the surface would distribute electrons and form equal quantities of two types of hydroxyl groups. A terminal OH is bound to a surface Ti^4+^ site, which has five coordinates with respect to the lattice oxide ions, and a bridging OH is bound to a Ti^4+^ site, which has four coordinates with respect to the lattice oxide ions [4]. Bridging OH groups should be strongly polarized by cations and, therefore, be acidic (pKa 2.9), while the terminal OH groups could be predominantly basic (pKa 12.7) [19,20,21]. Recently, it was reported that the pre-adsorbed water on the surface bridging hydroxyls could significantly modulate the surface property of TiO_2_ [22,23,24]. This water-mediated treatment may switch the adsorption mode of dyes and promote the photocatalytic efficiency. UV irradiation of TiO_2_ dispersion in water was also found to increase the surface bridging hydroxyls [25]. Nevertheless, the increase of acidic groups lowered the isoelectric point and reduced the positive charges of TiO_2_, leading to particle aggregation. The aggregation of TiO_2_ nanoparticles (NPs) in aqueous solution is very troublesome, and may not only decrease the photocatalytic properties but also hinder their application in the biomedical field, such as biological labeling and drug delivery [26,27,28]. Considering that the terminal hydroxyl group is basic and hydrophilic, the increase of terminal hydroxyls on the surface of TiO_2_ may increase the isoelectric point, cause more positive charges on the surface, and retard the particle aggregation. Therefore, it would be greatly desirable to prepare TiO_2_ NPs with abundant surface hydroxyl groups, particularly terminal hydroxyls.

The mixed H_2_O_2_ and NaOH solution, called alkaline hydrogen peroxide (AHP), has been used to improve the performance of commercial TiO_2_ powder while still remaining its morphology and crystalline structure [29]. In the present study, we confirmed that AHP treatment is a simple and economic strategy to prepare TiO_2_ NPs with abundant surface hydroxyl groups, especially terminal hydroxyls. The structure and morphology of AHP-treated TiO_2_ NPs were characterized by X-ray diffraction (XRD), atomic force microscopy (AFM), and dynamic light scattering (DLS). The surface hydroxyl groups were quantified by thermogravimetric analysis (TGA), Fourier transform infrared spectroscopy (FTIR), and X-ray photoelectron spectroscopy (XPS). The AHP-treated TiO_2_ NPs exhibit superior water-dispersibility and highly efficient dye sensitization without any complicated and expensive surface modification.

## 2. Materials and Methods

### 2.1. Materials

Titanium dioxide (ST-01) was purchased from Ishihara Sangyo Kaisha, Ltd. (Osaka, Japan). Alkaline hydrogen peroxide-treated TiO_2_ nanoparticles (denoted as AHP-TiO_2_ NPs or AHP-ST-01) were prepared using ST-01 powder as described previously [29]. Hydrogen peroxide solution (30% H_2_O_2_), sulforhodamine B (SRB), and 2,4-dinitrophenylhydrazine (>97%) were from Sigma-Aldrich. FeCl_3_·6H_2_O (>99.0%) and 1,10-phenanthroline hydrochloride monohydrate (>99.5%) were from Merck. Oxalic acid (C_2_H_2_O_4_·2H_2_O, >99.5%) was from Riedel-de-Haën. Sodium hydroxide (>98.9%) and hydrochloric acid (36.5–38%), methanol, and acetonitrile were from J.T. Baker. Doubly de-ionized water prepared with a Milli-Q system (Millipore, Bedford, MA, USA) was used exclusively for all solutions (≥18.2 MΩ-cm resistivity).

### 2.2. Characterization

The crystallization behavior of the AHP-TiO_2_ NPs was examined by X-ray diffraction (XRD) using Bruker D8X (Karlsruhe, Germany) with CuKα radiation (λ = 0.15406 nm) over the range 20° < 2θ < 80°. The surface morphology of the TiO_2_ NPs coated on glass was investigated by tapping-mode atomic force microscopy (AFM, Bruker Dimension Icon, Karlsruhe, Germany). Images were minimally processed and analyzed using NanoScope Analysis 1.5. X-ray photoelectron spectroscopy (XPS) analyses were performed on a PHI Quantera SXM spectrometer (ULVAC-PHI Inc., Kanagawa, Japan) using a monochromotized aluminum anode X-ray source (AlKα 1486.6 eV). The base pressure in the XPS analysis chamber during spectral acquisition was less than 10^−8^ Torr. All binding energy values were calibrated by fixing the C 1s line to 285 eV. Peak deconvolutions were performed using Gaussian-Lorentzian components with identical full width at half maximum (FWHM) parameters after a Shirley background subtraction. The elemental composition of the samples was determined using peak area ratio and the sensitivity factor (SF) of each element in XPS. The Brunauer-Emmet-Teller (BET) surface areas of powders were evaluated from nitrogen adsorption and desorption isotherms at 77 K, recorded by using a Micromeritics ASAP 2020 nitrogen adsorption apparatus. Thermogravimetric analysis (TGA) was carried out on a Mettler Toledo STARe ThermoGravimetric Analyzer (TGA/SDTA851e, Mettler Toledo, Columbus, OH, USA). All measurements were taken under a constant flow of nitrogen of 40 mL/min. The temperature was first increased from room temperature to 120 °C (T_1_), held at this temperature for 10 min to remove the physically adsorbed water, and then increased from 120 to 500 °C (T_2_) at a rate of 20 °C/min. The OH surface density of TiO_2_ powders was calculated using the TGA weight loss and the specific surface area (SSA) by the following equation [30,31]:

(1) $$number\;OH/nm^{2}=\alpha \frac{{\left(\mathrm{number}\;\mathrm{OH}/{\mathrm{nm}}^{2}\right)}_{T_{2}}\times \mathrm{SSA}\times {\mathrm{wt}}_{T_{2}}+\left[\frac{{\mathrm{wt}}_{T_{1}}-{\mathrm{wt}}_{T_{2}}}{\frac{{\mathrm{MW}}_{H_{2}O}}{N_{A}}}\times 2\right]}{\mathrm{SSA}\times {\mathrm{wt}}_{T_{1}}}$$

where SSA is the specific surface area, wt_T_1__ and wt_T_2__ are the sample weights at the corresponding temperature T_1_ and T_2_ , respectively, MW_H_2_O_ is the molecular weight of water, N_A_ is Avogadro’s constant, and α is a calibration factor. The value of α is given as 0.625. It is assumed that 120 °C is the proper temperature to separate physically adsorbed and chemically bound water molecules and the surface of the TiO_2_ powders is free of OH surface groups at 500 °C. Dynamic light scattering (DLS) and zeta potential measurements of the dispersions were performed using a Malvern Zetasizer Nano ZS instrument (ZEN 3600, Malvern Instruments, Southborough, MA, USA) equipped with an He-Ne laser at a wavelength of 633 nm and a back-scattering detection angle of 173°. For batch mode hydrodynamic size (diameter) measurements, samples prepared for the DLS measurements were loaded into standard path-length 10 mm disposable optical polymethyl methacrylate (PMMA) cuvettes followed by equilibration (typically 5 min) to 25 °C. For zeta potential measurements, samples were loaded into a pre-rinsed folded capillary cell and an applied voltage was automatically set by the software. A minimum of three measurements were made per sample. Fourier transform infrared spectra (FTIR) were recorded on a Nicolet 6700 FT-IR spectrometer (Thermo Electron Scientific Instruments, Madison, WI, USA). Spectra were scanned over the range of 400–4000 cm^−1^. All of the dried samples were mixed with KBr and then compressed to form pellets. Ultraviolet—visible absorbance and transmittance measurements were made with a Varian Cary 50 spectrophotometer (Varian, Palo Alto, CA, USA) and a custom-built constant-temperature (25 °C, BL-20 TIT recirculator) variable-path-length aluminum cuvette holder (black-anodized). The dispersible property of TiO_2_ NPs could be quantitatively evaluated by D_x_ % as follows:

(2) $$D_{x}\%=\frac{T_{0}}{T_{x}}\times 100\%$$

where D_x_ % was the transmittance change of the dispersion, T_0_ and T_x_ were the transmittances of fresh TiO_2_ dispersion and the TiO_2_ colloidal solution after setting for x hours, respectively [26]. The photoluminescence (PL) spectra were made with a Varian Cary Eclipse fluorescence spectrophotometer (Varian, Palo Alto, CA, USA).

### 2.3. Light Source and Photocatalytic Efficiency

An illumination system (Oriel) was constructed from a 1000 W Xe arc lamp (ORC), an F/1 UV grade fused silica condenser, a water filter with quartz windows (6 cm, Oriel 6123), a shutter, focusing lens assembly from Spectral Energy (Kratos), two 2.5 mm Hoya Y-48 optical glass filters, and a reaction cell. The two cutoff filters were placed to completely remove any radiation below 480 nm and to ensure illumination by visible light only (Figure S1 in the Supplementary Materials). The reaction cell was a semi-cylindrical shape with an optically flat quartz window fused to the pyrex cell and at right angles to the collimated light beam as described previously [29]. The volume-averaged incident irradiance I_0_ in the range of 480–550 nm was determined by ferrioxalate chemical actinometry [32,33,34]. It should be noted that the quantum yield and molar absorptivity of ferrioxalate actinometer are closed to zero beyond 550 nm. Values of the volume-averaged incident irradiance I_0_ ranged from 5.21 to 11.5 μ(einstein)L^−1^s^−1^ for these experiments.

The aqueous colloid dispersion (100 mL) with a certain amount of SRB and photocatalyst was placed in a vessel. Prior to irradiation, the dispersion was magnetically stirred in the dark for about 30 min to establish an adsorption-desorption equilibrium. At a predetermined time interval, 1.5 mL solution was withdrawn and centrifuged to separate particles. The degradation rate of SRB was determined by monitoring the decrease in its maximum absorbance at 563 nm.

### 2.4. Determination of Hydroxyl Radical Species

The quantity of hydroxyl radicals in different TiO_2_ aqueous dispersions could be estimated by the oxidation of methanol to formaldehyde [35,36]. The amounts of formaldehyde formed in the reactions were determined by HPLC using 2,4-dinitrophenylhydrazine (DNPH) derivatization [37]. At regular time intervals, 1.5 mL solution was withdrawn and passed through a 0.22 µm polyvinylidene difluoride (PVDF) membrane filter to remove particles. Twenty-five µL of 10 mM DNPH derivatizing reagent was then added in the sample solution and pH was adjusted to 1.7 by adding concentrated hydrochloric acid. The vial was capped and shaken slowly, and the reaction was allowed to proceed for 30 min at 25 °C before injection. The binary gradient HPLC system consisted of the following components connected in series: a microvolume double-plunger pump (LC-20AT, Shimatzu, Tokyo, Japan), a Rheodyne (Cotati, CA, USA) Model 7125 injector with 100 µL loop, a guard column, a Beckman Ultrasphere C18 column (150 mm × 4.6 mm; 5 µm particle size), and a Spectra-Physics SP-8450 UV/vis detector (Spectra-Physics, Inc., Santa Clara, CA, USA) set at 360 nm. The data were collected and processed by a chromatography data system (SISC 32.3.1, New Taipei City, Taiwan).

## 3. Results and Discussion

### 3.1. Characterization of AHP-Treated TiO_2_ NPs

**Figure 1:**
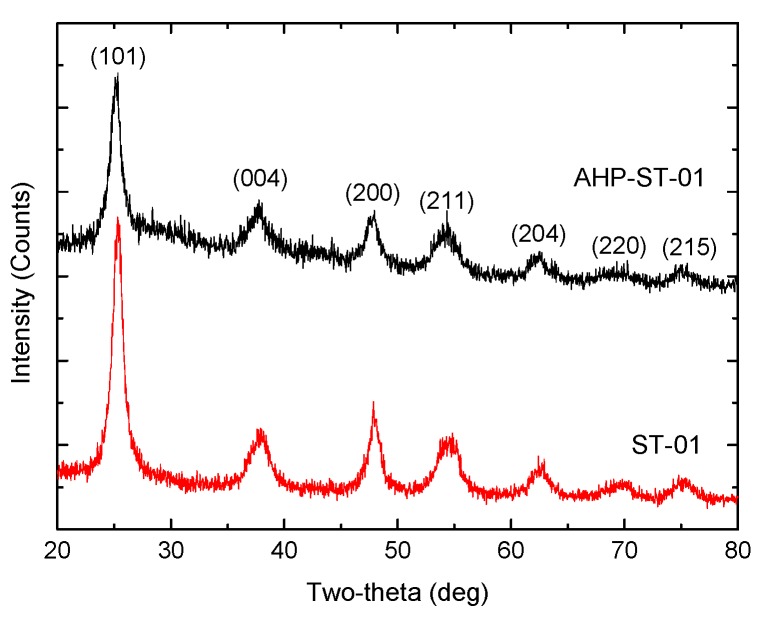
XRD spectra of TiO_2_ powders: ST-01 and AHP-ST-01.

The crystal structure, morphology, and agglomeration of untreated and AHP-treated TiO_2_ NPs were investigated. The XRD patterns of ST-01 and AHP-ST-01 show peaks attributed to (101), (004), (200), (211), (204), (220), and (215) reflections of the anatase (Figure 1). No diffraction peaks due to other phases (for example, rutile or brookite) were observed. It indicates that the TiO_2_ crystal phase was not changed after AHP pretreatment. The average particle sizes estimated from the Scherrer equation were 7 nm for untreated ST-01 and 5 nm for AHP-ST-01. The decrease in size was ascribed to the corrosion of AHP treatment and the result could be further confirmed by direct AFM observation.

**Figure 2:**
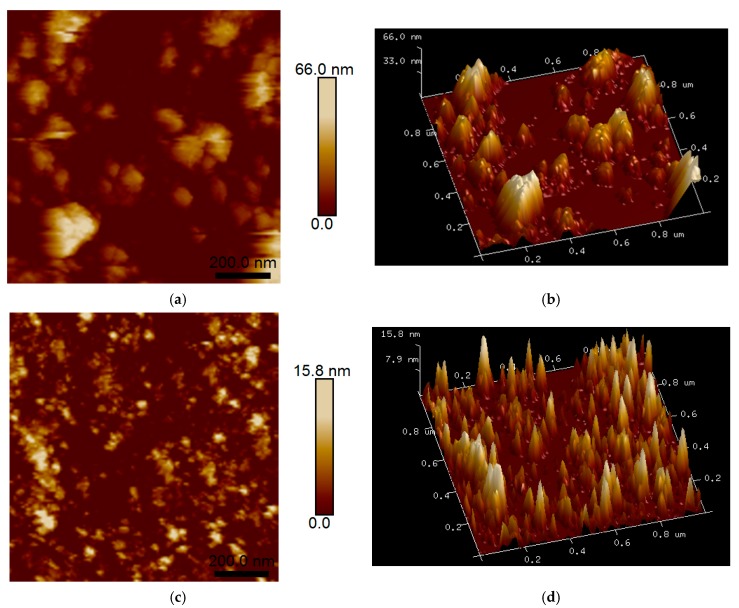
AFM images of TiO_2_ powders: (**a**,**b**) ST-01 and (**c**,**d**) AHP-ST-01.

Figure 2 depicts two and three-dimensional AFM images of untreated ST-01 and AHP-ST-01 on the glass slide. As can be seen, the surface morphology and roughness revealed a significant difference between untreated and AHP-treated TiO_2_ NPs. Untreated TiO_2_ NPs were aggregates of many small particles into around 66 nm spheres. In contrast, AHP-TiO_2_ NPs were evenly distributed on the glass substrate and showed well-separated primary particles. The distribution behavior of AHP-TiO_2_ NPs is probably attributable to the enhanced surface hydroxyl groups, which can help it to anchor on the surface of glass and improve the adhesion quality of the coatings. The shape of AHP-TiO_2_ NPs was sphere, indicating that the formation mechanism of TiO_2_ NPs by AHP treatment is different from that of TiO_2_ nanotubes by hydrothermal treatment [38].

**Figure 3:**
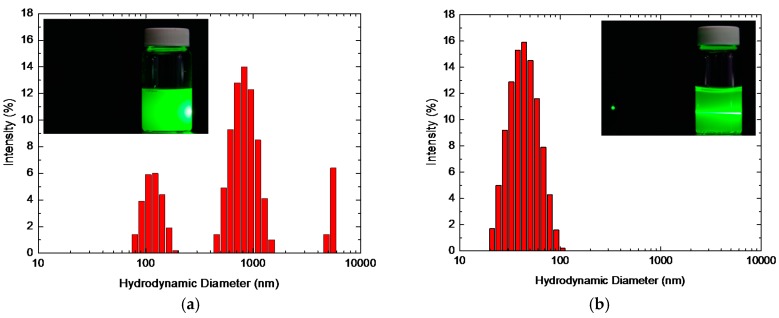
Particle size distributions of (**a**) ST-01 and (**b**) AHP-ST-01 NPs in aqueous solution. [TiO_2_] = 1.5 g/L. Insets: photographs of ST-01 and AHP-ST-01 suspension where a green laser beam passes through.

DLS permits the determination of the particle size distribution from dispersed TiO_2_ NPs in water. As shown in Figure 3, untreated ST-01 was significantly aggregated in water with an average size of 820 nm, and AHP-ST-01 was dispersible in water with an average size of 40 nm. Since AHP treatment could prevent TiO_2_ particle aggregation and reduce light scattering, a green laser beam passed through AHP-ST-01 dispersed easily. The obtained average particle sizes are considerably larger than that of the dried TiO_2_ NPs due to the contribution of the hydration layer and the inherent agglomeration of TiO_2_ NPs in water [39]. Table 1 lists the physicochemical properties of Degussa P25, ST-01, and AHP-ST-01, including specific area, hydrodynamic size, zeta potential, isoelectric point, and amount of surface OH groups [40].

The amount of surface OH groups was determined by TGA, FTIR, and XPS. Compared to the other techniques, TGA is a simple, fast, and reliable method for determining surface OH groups of titania powders [31,41]. The TGA weight loss indicates the removal of adsorbed water and the TGA curves are shown in Figure S2. Two clear steps in weight loss at 120 °C are present in relation to the physically adsorbed water and chemically bound water molecules [30]. According to the TGA analysis, it appears that AHP-ST-01 has much more surface OH groups than any other untreated samples. AHP treatment can enhance the total amount of surface OH groups of ST-01 from 2.32 to 6.08 nmol/g. A study demonstrated that the losses in the 120–300 °C and 300–600 °C ranges can be related to weakly bonded OH groups and strongly bonded OH groups, respectively [41]. The weight loss of AHP-ST-01 at high temperature is still significant and can be attributed to the loss of water that is produced by the condensation of neighboring terminal OH groups [42]. The weakly bonded OH groups and strongly bonded OH groups of ST-01 and AHP-ST-01 have been distinguished and listed in Table 2.

The FTIR spectra of TiO_2_ powders from ST-01, Degussa P25, and AHP-ST-01 are shown in Figure S3. A broad band in the region of 3700–2500 cm^−1^ with a maximum at 3440 cm^−1^ is attributed to the OH stretching vibrations [10,43,44]. As can be seen, the peak area in the absorption band for AHP-ST-01 was increased, implying that AHP-ST-01 contains more surface OH groups than untreated ST-01. Furthermore, the band with a blue shift was associated with the formation of terminal OH groups [44]. The relative amounts of surface OH groups were normalized to Degussa P25 as follows: Degussa:ST-01:AHP-ST-01 = 1:1.9:3.2, listed in Table 1. However, it should be noted that the water adsorbates observed under the vacuum were very strongly bound through oxygen of water to a less coordinated Ti cation and a hydrogen bond to a surface oxygen atom [8]. In the FTIR study of ST-01 at 0.01 Pa, 80% of the intensity of the OH stretching band at room temperature was contributed by molecularly adsorbed water, while only 20% was by surface OH. By taking account of the effects of hydrogen bonding, surface OH groups exhibit a strong interaction with adsorbed water molecules [20]. Thus, there is only indirect evidence that the absorption band is qualitatively proportional to the amount of surface OH groups.

**Figure 4:**
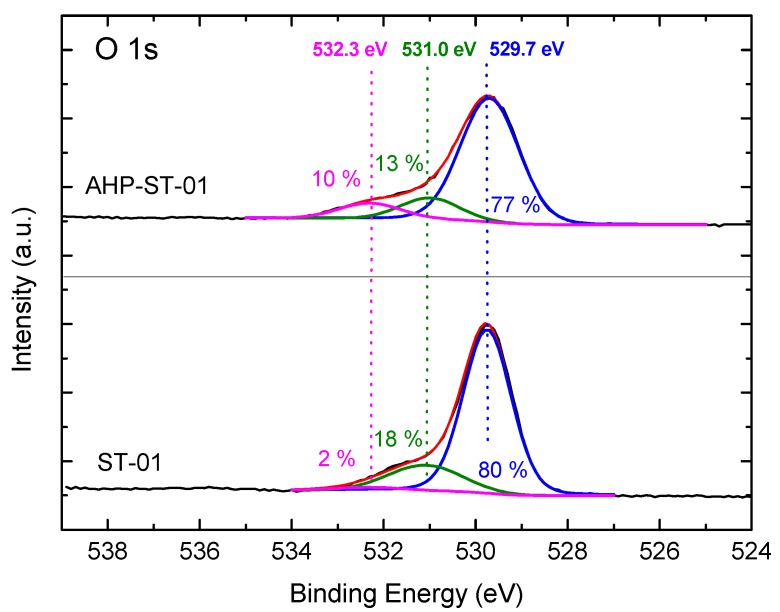
O 1s XPS spectra of ST-01 and AHP-ST-01. The relative ratios of the peak areas are shown.

Figure 4 illustrates the high resolution XPS spectra of O 1s core levels of TiO_2_ samples before and after AHP treatment. The peak deconvolution indicated the presence of three Gaussian component peaks located at 529.7, 531.0, and 532.3 eV, corresponding to Ti-O in surface bulk oxide lattices (denoted by TiO_2_), acidic bridging OH groups, and physisorbed H_2_O (denoted by TiOH) with basic terminal OH groups (denoted by Ti-OH), respectively [11,19,45,46]. As shown in Figure 4, the major OH species is supposed to be the bridging OH groups on ST-01, similar to previous observation [20]. After AHP treatment, the intensity of the shoulder at 532.3 eV corresponding to the Ti-OH terminal group increased dramatically.

**Scheme 1:**
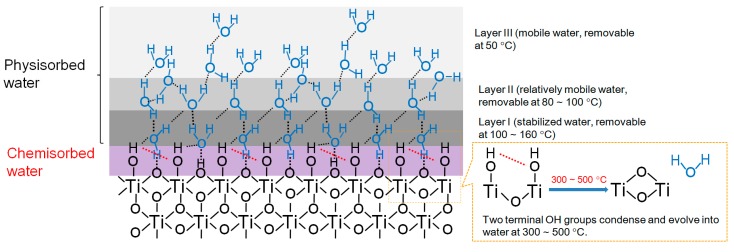
Pictorial representation of proposed structure of water layers adsorbed on the surface of TiO_2_ NPs.

The concentration and relative amount of surface OH species on ST-01 before and after AHP treatment are presented in Table 2. Comparison of the results from TGA with XPS analysis did reveal surprisingly consistent changes in the amount of terminal OH groups after AHP treatment. Both measurements indicated a markedly enhanced OH surface density of TiO_2_ NPs after AHP treatment. A comparison of surface OH groups of different TiO_2_ NPs is given in Table 3 [47,48]. As shown in Table 3, AHP-ST-01 has dramatically enhanced the OH surface density to 12.0 OH/nm^2^, which is an unprecedented value. The density is even comparable with that of three-dimensional TiO_2_ nanostructure with needlelike surface synthesized by a special self-assembled method [15]. As mentioned previously, a simple and green way to fabricate TiO_2_ NPs with uniform particle size and high surface hydroxyl groups is absolutely required for industrial production. On the basis of the results of this study and the literature [20,43,49,50,51,52,53,54,55,56], a pictorial representation of the structure of water layers adsorbed on the surface of TiO_2_ NPs is proposed in Scheme 1.

**Figure 5:**
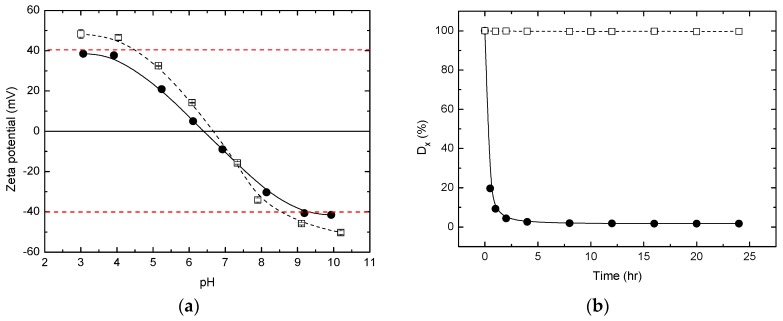
(**a**) Zeta potentials of ST-01 (●) and AHP-ST-01 (□) NPs suspended in water with mass concentration of 0.5 g/L as a function of pH; (**b**) Variation of the transmittance change (D_x_ %) of ST-01 (●) and AHP-ST-01 (□) NPs in aqueous suspension after setting for x h. [TiO_2_] = 0.5 g/L, pH = 3.

**Figure 6:**
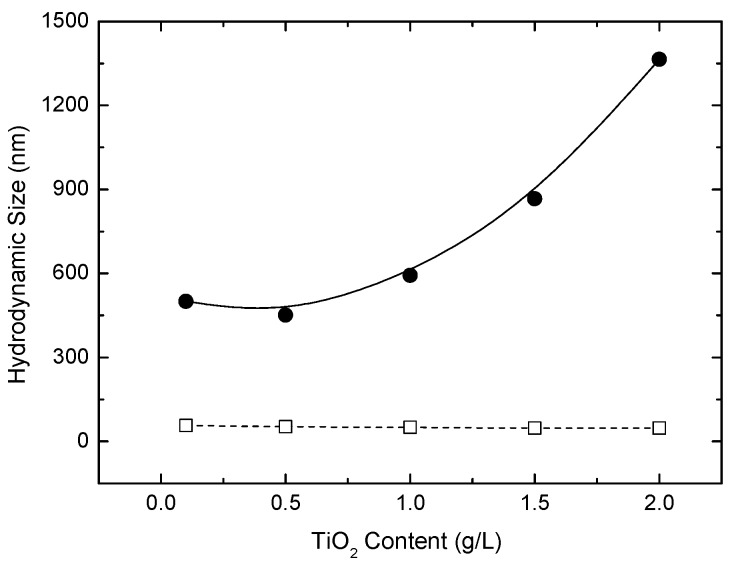
Effect of TiO_2_ content on hydrodynamic size for ST-01 (●) and AHP-ST-01 (□) NPs in aqueous suspension.

Figure 5a shows the zeta potentials of TiO_2_ NPs suspended in water. AHP treatment increased the isoelectric point of ST-01, so the terminal OH groups on the TiO_2_ surface were increased. The zeta potential of AHP-ST-01 changed from 48 mV to −50 mV when adjusting pH from 3 to 10. The high zeta-potential of the AHP-ST-01 reveals their high surface charge given by the surface groups, which could retard the particle aggregation. Figure 5b shows that the transmittance change of AHP-ST-01 dispersion remained at 99.7% for long periods of time, suggesting its better dispersive property in water. The effect of TiO_2_ concentration on the aggregation of TiO_2_ NPs was examined. As shown in Figure 6, hydrodynamic sizes of untreated ST-01 dispersion increased with increasing TiO_2_ concentration from 0.1 to 2.0 g/L. In contrast, the hydrodynamic sizes of AHP-ST-01 were insensitive to TiO_2_ content. These results were all consistent with previous analysis showing that terminal hydroxyls were formed abundantly, which increased the isoelectric point and the positive charges of TiO_2_ leading to enhanced water dispersibility.

### 3.2. Photocatalytic Activity

**Figure 7:**
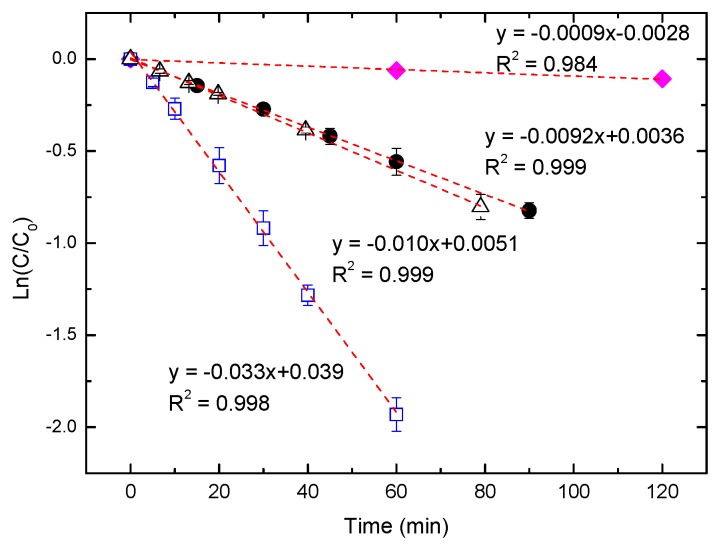
Pseudo-first-order kinetic fits for visible-light photodegradation of SRB without TiO_2_ catalyst (♦) and in different colloidal solutions for ST-01 (●), ST-01 pretreated in water for 36 h. (Δ), and AHP-ST-01 (□). [TiO_2_] = 1.5 g/L, [SRB] = 10 µM, pH = 3.

**Figure 8:**
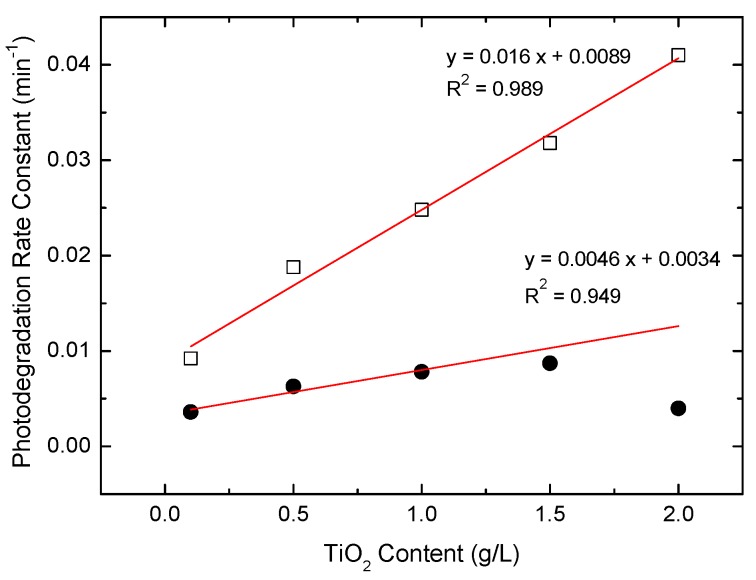
Effect of TiO_2_ content on the photocatalytic degradation of SRB under visible light irradiation at pH 3 for ST-01 (●) and AHP-ST-01 (□) NPs. Linear regression fit was plotted through first three data points for untreated ST-01 suspension.

Figure 7 shows that the visible-light photocatalytic degradation rates of SRB over TiO_2_ followed pseudo-first-order kinetics. The original changes in the absorption spectra for SRB at various irradiation time intervals are shown in Figures S4–S6. The dye concentration remained almost constant for 2 h under visible light without TiO_2_ as a control experiment. The rate constants exhibited the following trend: AHP-ST-01 >> water-treated ST-01 > ST-01. AHP-ST-01 had a four times higher rate constant than did ST-01, which indicated that AHP treatment was much more efficient for the promotion of dye sensitization of TiO_2_ than water-treatment. This trend could be mainly attributed to the amount of adsorption of the as-prepared TiO_2_ for SRB [29,57]. In the previous studies, the visible-light photocatalytic activity was highly correlated with the adsorption capacity because a preliminary adsorption of substrate on the catalyst surface was a prerequisite for highly efficient dye sensitization. In acidic conditions, the positively charged TiO_2_ surface strongly attracts anionic SRB, so the photodegradation rate of SRB can be greatly enhanced due to the increase in the adsorbed amount of SRB (Figure S7). As dye concentration increases, some dissociative dye molecules, which cannot participate in electron transfer on the TiO_2_ surface but absorb a part of incident light at the same time, would also increase and result in a decrease of the photodegradation rate by a solution filter effect (Figures S8 and S9). Because of the superior water-dispersibility of AHP-ST-01, the photodegradation rate could be proportional to the amount of TiO_2_ up to a high concentration at 2 g/L as shown in Figure 8. In such high TiO_2_ concentrations, the assumption of active sites as proportional to the quantity of TiO_2_ was still valid.

**Figure 9:**
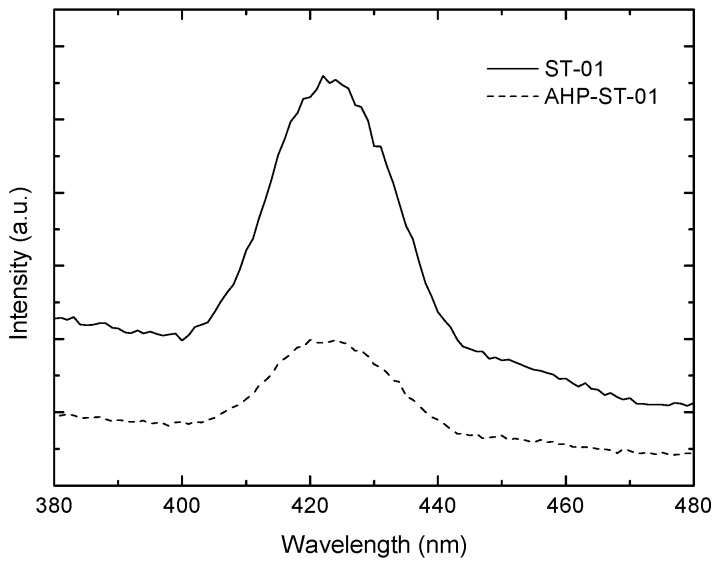
Photoluminescence spectra of ST-01 (solid line) and AHP-ST-01 (dash line) NPs dispersed in ethanol. [TiO_2_] = 0.2 g/L, pH = 3.

**Figure 10:**
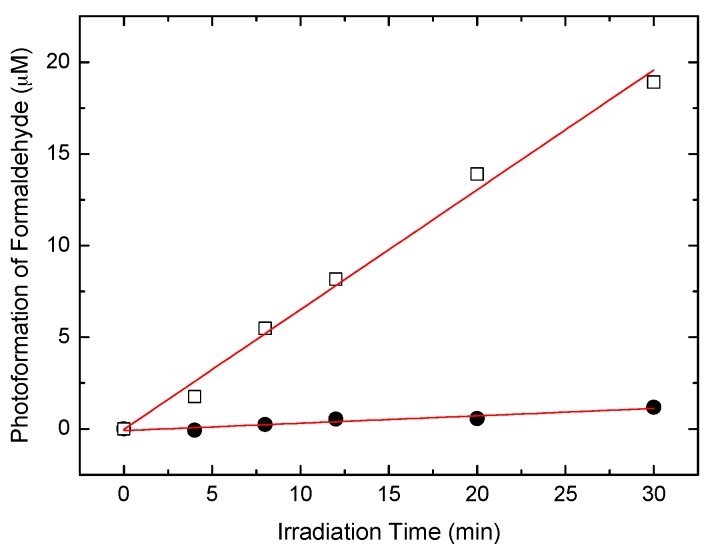
Photoformation of formaldehyde in ST-01 (●) and AHP-ST-01 (□) aqueous suspensions under visible light irradiation. [TiO_2_] = 0.5 g/L, [SRB] = 10 µM, [CH_3_OH] = 1 M, pH = 3.

These results indicated that high surface OH groups of TiO_2_ NPs could not only reduce particle aggregation and maintain their photoactivity in high concentrations but also inhibit the electron-hole recombination, which has been confirmed by photoluminescence (PL) (Figure 9). The behaviors of the photodegradation rate were also consistent with the measurements of hydroxyl radical species estimated by the oxidation of methanol to formaldehyde, shown in Figure 10.

### 3.3. Formation of TiO_2_ NPs with High Surface Hydroxyl Groups

**Scheme 2:**
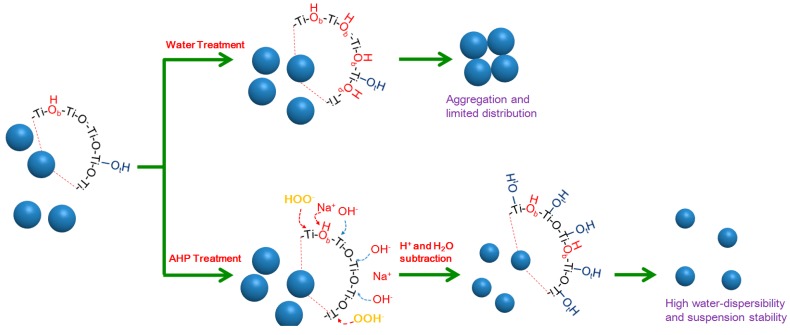
Comparison of the formation of hydroxyl groups on the surface of TiO_2_ NPs pretreated by water and alkaline hydrogen peroxide.

A comparison of the preparation of TiO_2_ NPs by water treatment and alkaline hydrogen peroxide treatment is illustrated in Scheme 2. Although water-mediated treatment may modulate the surface electronic structure of TiO_2_, switch the adsorption mode of dyes, and promote dye sensitization, the increased bridging hydroxyls on the surface reduce the positive charges of TiO_2_ and cause particle aggregation, negatively affecting catalytic performance. Unlike water treatment, AHP treatment can effectively corrode the TiO_2_ surface, reduce the particle size, and increase the surface OH groups, particularly terminal hydroxyls. This treatment can not only enhance the water-dispersibility by increasing the surface potentials but also promote the dye sensitization process by generating more hydroxyl radicals. Both advantages could be attributed to the substantially increased terminal hydroxyls on the surface [15,16]. Due to the high water-dispersibility and dispersion stability, AHP-TiO_2_ NPs have a great potential use for fabrication of coatings, self-cleaning, dye sensitized solar cells, drug delivery, etc.

## 4. Conclusions

In this study, we have shown that AHP treatment is a simple and green way to improve the performance of commercial TiO_2_ powder, which could be used for mass production of TiO_2_ NPs with uniform particle size and high surface hydroxyl groups. Based on TGA, FT-IR, and XPS analysis, the AHP-treated TiO_2_ NPs have a high density of hydroxyl groups, approximately 12.0 OH/nm^2^, which is an unprecedented result. The increase of terminal hydroxyls on the TiO_2_ surface can not only enhance the water-dispersibility but also promote dye sensitization. The highly water-dispersible TiO_2_ NPs in water without adding any stabilizing ligand may have widespread uses in environmental, energy, and biomedical applications.

## Figures and Tables

**Table 1:** Physicochemical properties of different TiO_2_ NPs.

Sample	Surface Area *^a^* (m^2^/g)	Crystalline Size *^b^* (nm)	Hydrodynamic Size *^c^* (nm)	Zeta Potential *^d^* (mV)	pH_IEP_	Surface OH *^f^* (mmol/g)	Relative Amount of Surface OH *^g^*
Degussa P25	56	20	259 ± 5	35 ± 5 *^e^*	6.3 *^e^*	0.49	1
Ishihara ST-01	290	7	821 ± 97	38 ± 1	6.3	2.32	1.9
AHP-ST-01	304	5	41 ± 1	48 ± 2	6.7	6.08	3.2

*^a^* BET specific surface area by N_2_ adsorption method; *^b^* Calculated from the XRD lines widths; *^c^* DLS analysis (*n* = 3); *^d^* Measured at pH 3; *^e^* Value reported from reference (Ryu and Choi, 2008) [40]; *^f^* Calculated from thermogravimetric analysis (TGA); *^g^* The relative amounts of surface OH were proportional to the peak areas of the OH stretching in the region of 3700–2500 cm^−1^ and were normalized to Degussa P25. The peak areas were calculated by absorbance spectra converted from the transmittance ones of FTIR.

**Table 2:** Concentration and relative amount of surface OH groups determined by TGA and XPS.

Sample	TGA			XPS		
	OH_weak_ (mmol/g)	OH_strong_ (mmol/g)	Relative Amount of OH_strong_	TiOH *^a^* (mmol/g)	Ti-OH *^b^* (mmol/g)	Relative Amount of Ti-OH
ST-01	1.73	0.59	1	4.67	0.51	1
AHP-ST-01	2.47	3.61	6.1	3.62	2.74	5.4

*^a^* TiOH: bridging hydroxyl group; *^b^* Ti-OH: terminal hydroxyl group.

**Table 3:** Comparison of surface OH groups of different TiO_2_ NPs.

Sample	S_BET_ (m^2^/g)	Surface OH Conc. (mmol/g)	Surface OH Density (/nm^2^)	Analytical Method	Reference
Degussa P25	56	0.44	4.8	TGA	Mueller et al., 2003 [30]
Degussa P25	56	0.49	5.3	TGA	This work
Hombikat UV 100	337	1.15	2.5	NH_3_-TPD	Carneiro et al., 2010 [47]
Kanto	22	0.012	0.3	Ion-Exchange	Tamura et al., 1999 [48]
TiO_2_ by sol-gel	85	1.44	10.2	TGA	Zhao et al., 2008 [31]
Three-dimensional TiO_2_ by sol-gel	203	4.01	11.9	XPS	Guo et al., 2013 [15]
NaCl-TiO_2_ by sol-gel	— *^a^*	2.31	—	TGA	Wang et al., 2012 [9]
NaOH-Degussa P25	—	1.60	—	TGA	Eskandarloo et al., 2015 [17]
Ishihara ST-01	290	2.32	4.8	TGA	This work
AHP-ST-01	304	6.08	12.0	TGA	This work

*^a^* No information available.

## Supplementary Materials

Nine figures related to this article are available online at www.mdpi.com/1996-1944/10/5/566/s1.
