# Supplementary material for: Markedly Enhanced Surface Hydroxyl Groups of TiO2 Nanoparticles with Superior Water-Dispersibility for Photocatalysis
Source: Materials (Basel). 2017 May 22;10(5):566. doi: 10.3390/ma10050566 (PMC5459044; doi:10.3390/ma10050566)
Supplement: Supplementary file 1 [file materials-10-00566-s001.pdf]

Electronic Supplementary Materials

Markedly Enhanced Surface Hydroxyl Groups of TiO_2 Nanoparticles with Superior Water-Dispersibility for Photocatalysis

Chung-Yi Wu [1], Kuan-Ju Tu [1], Jin-Pei Deng [2], Yu-Shiu Lo [1] and Chien-Hou Wu [1,*]

[1] Department of Biomedical Engineering and Environmental Sciences, College of Nuclear Science, National Tsing Hua University, Hsinchu 30013, Taiwan; s9812805@m98.nthu.edu.tw (C.-Y.W.); sacredbless@hotmail.com (K.-J.T.); yslo2001@gmail.com (Y.-S.L.)

[2] Department of Chemistry, Tamkang University, Taipei 25137, Taiwan; jpdeng@mail.tku.edu.tw

* Correspondence: chwu@mx.nthu.edu.tw; Tel.: +886-3-571-5131 (ext. 35856)

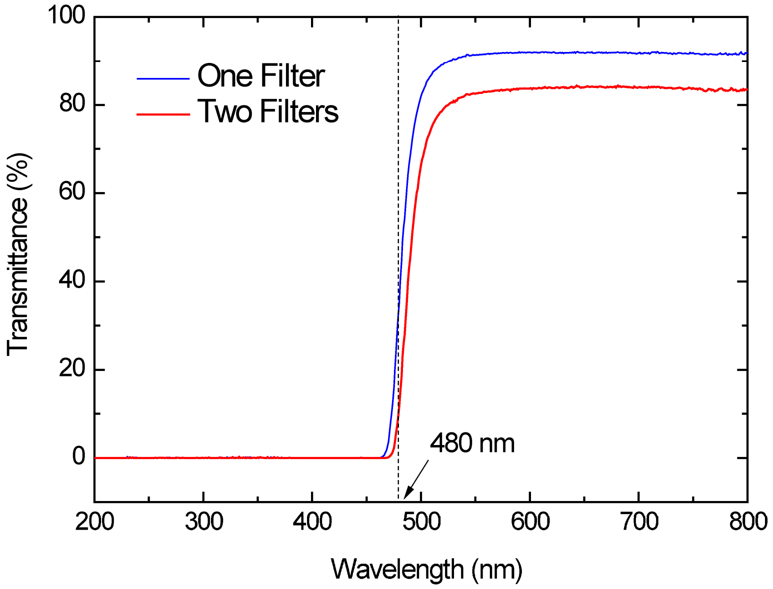

Figure S1. Transmittance spectra of 2.5-mm Hoya Y-48 optical glass filter (Hoya Corp. USA) with wavelength. The Hoya Y-48 optical filter is a sharp cut filter that cuts off as much as possible of the wavelength light shorter than 480 nm while transmitting as much of longer wavelength light as possible. The two yellow cutoff filters were placed to completely remove any radiation below 480 nm and to ensure illumination by visible light only.

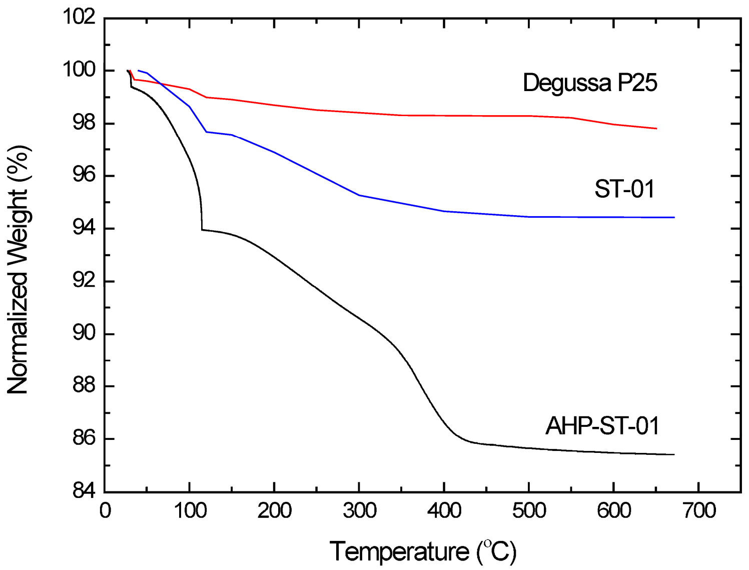

Figure S2. TGA curves of TiO2 samples: Degussa P25, ST-01, and AHP-ST-01.

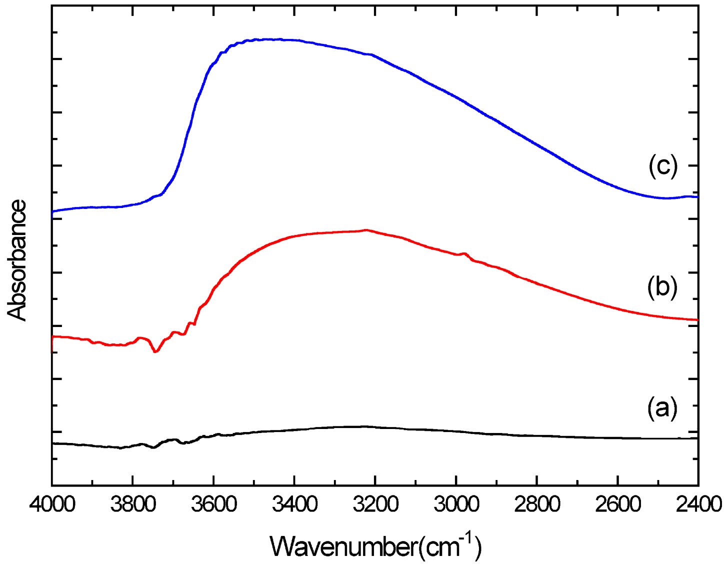

Figure S3. FT-IR spectra of TiO2 powders: (a) untreated TiO_2 (Degussa P25), (b) untreated TiO_2 (ST-01), and (c) AHP-treated TiO_2 (ST-01). All samples were dried at 60 °C for 12 h and milled into tiny powders for measurements.

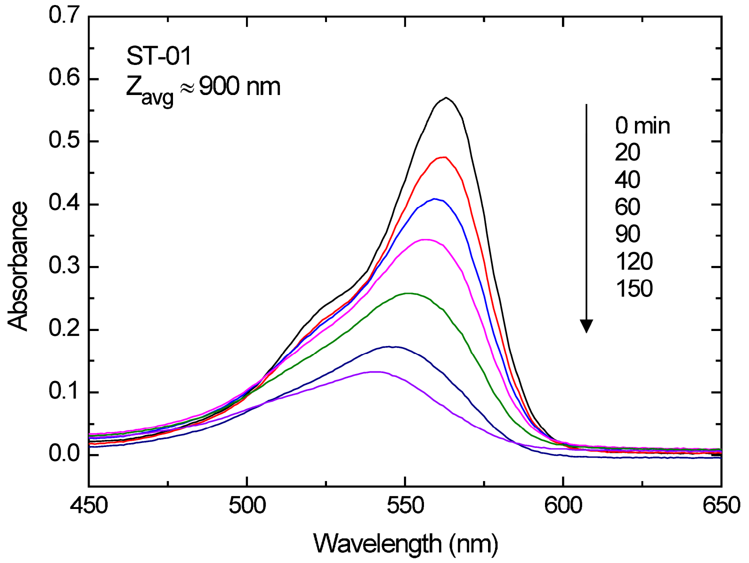

Figure S4. Absorption spectra of SRB solutions (10 μM) at various irradiation times in the presence of ST-01. [TiO_2] = 1.5 g/L, [SRB] = 10 μM, pH = 3.

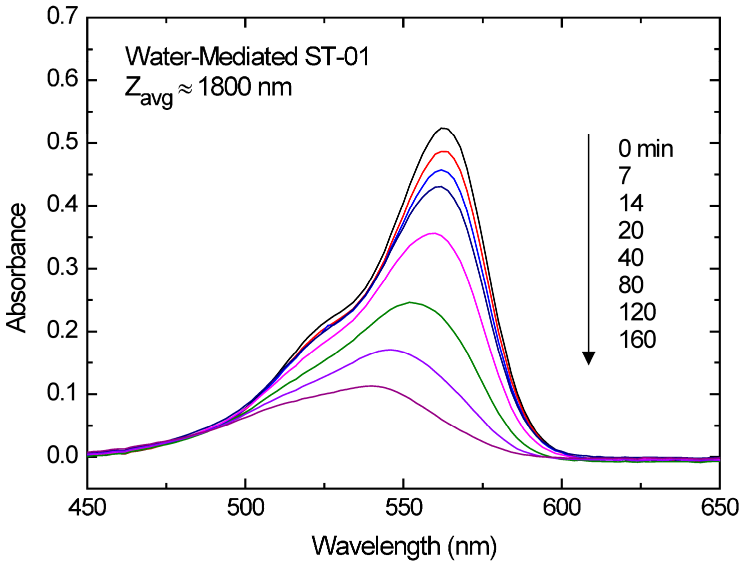

Figure S5. Absorption spectra of SRB solutions (10 μM) at various irradiation times in the presence of ST-01 pretreated in water for 36 hrs. [TiO_2] = 1.5 g/L, [SRB] = 10 μM, pH = 3.

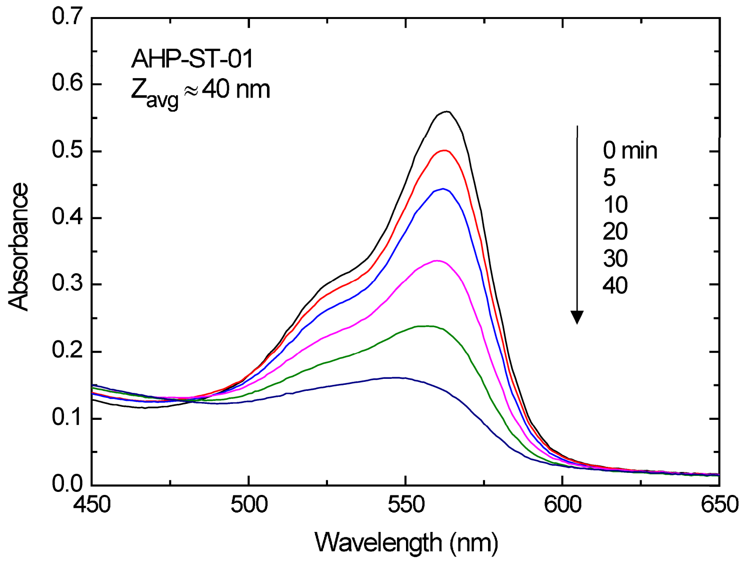

Figure S6. Absorption spectra of SRB solutions (10 μM) at various irradiation times in the presence of AHP-ST-01. [TiO_2] = 1.5 g/L, [SRB] = 10 μM, pH = 3.

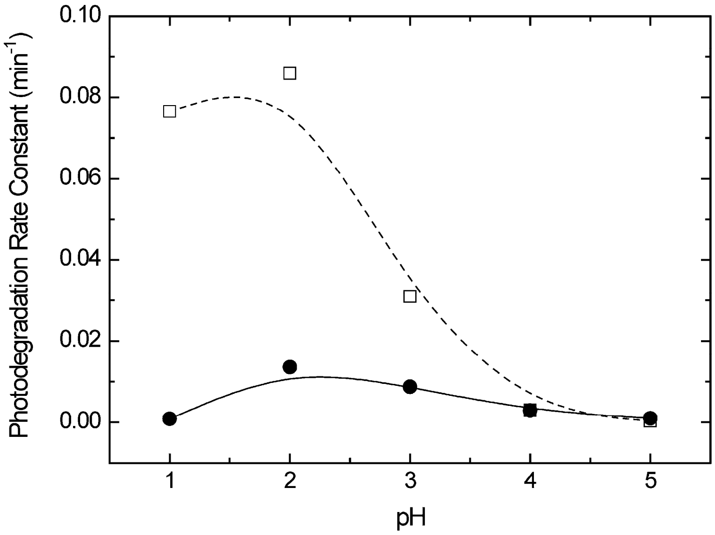

Figure S7. Photodegradation rate constant of SRB in ST-01 (●) and AHP-ST-01 (□) suspension as a function of pH. Light source: 1000 W Xenon lamp (λ > 480 nm), [TiO_2] = 1.5 g/L, [SRB] = 10 μM.

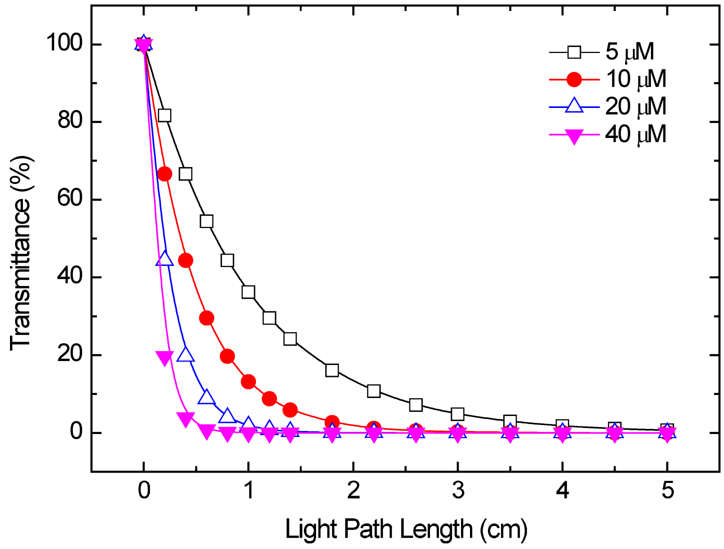

Figure S8. Effect of SRB concentration on the light transmittance at 563 nm where the maximum absorbance of SRB was obtained.

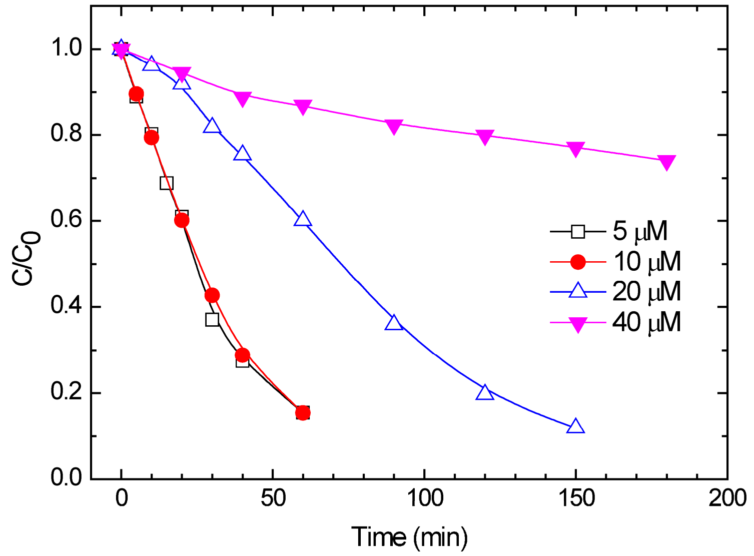

Figure S9. Photocatalytic degradation of SRB in aqueous suspension of AHP-TiO_2 NPs with different SRB concentrations under visible light irradiation (λ > 480 nm). [AHP-TiO_2] = 1.5 g/L, pH = 3.

© 2017 by the authors; licensee MDPI, Basel, Switzerland. This article is an open access article distributed under the terms and conditions of the Creative Commons by Attribution (CC-BY) license (http://creativecommons.org/licenses/by/4.0/).
